# 

**Published:** 1832

**Authors:** 


### CONTENTS.

### Essays and Cases.

ART. PAGE.

I.—An Inquiry into the Anatomical Condition of the Alimentary Canal in its healthy state. Extracted from the Pathological Anatomy of Professor Andral, - - - - 169

II.—On the Use of Digitalis in Amenorrhea, with cases of its successful administration. By W. T. S. Cornett, M. D. - - - 184

III.—Observations on the Modus Operandi of Calomel. By a Correspondent, - - - 189

IV.—Case of Complete Division of the Tendo Achillis, by the blow of an Axe. Communicated by John L. Richmond, M. D. of Cincinnati, - 191

V.—Case of Pregnancy and Labor attended with Convulsions, and complicated with many anomalous symptoms, terminating fatally. Reported by John L. Richmond, M. D. of Cincinnati, 194

VI.—Notices of the Epidemic Constitution of the Summer of 1832, at Cincinnati. By Daniel Drake, M. D. - - - - - 198

### Reviews and Bibliographical Notices.

VII.—A Practical Compendium of Midwifery, being the course of Lectures on Midwifery and on the Diseases of Women and Infants, delivered at St. Bartholomew's Hospital, by the late Robert Gooch, M. D. Philadelphia: Carey and Hart—pp. 120 —octavo—1832. - - - - 211

CONTENTS.

VIII.—A Treatise on the Venereal Diseases of the Eye. By W. M. Lawrence, F. R. S., late professor of Surgery to the Royal College of Surgeons in London, Surgeon to St. Bartholomew's Hospital, &c. - - - - - - 229
IX.—Surgical Memoirs of the Campaigns of Russia, Germany and France. By Baron D. J. Larrey, 244
X.—Epidemic Cholera, - - -

### Miscellaneous Intelligence.

1. Wound of the Head.—Ball extracted from the Brain, 272
2 Wound of the Head.—Severe Injury of the head, with Loss of Speech and other Nervous Affections, - 274
3. Wound of the Thorax.—Penetrating Sabre Thrusts, 277
4. Wound produced by the Discharge of a Pistol within the Cavity of the Mouth, - - - 278
5. Thoughts on Insanity, - - - - 280
6. Treatment of Gout, - - - - 282
7. Case of Iliac Aneurism with ligature of the External Iliac Artery, - - - - 285
8. Re-union of Divided Parts, - - - 288
9. Irreducible Hernia, - - - 289
10. Symptoms of Aortic Aneurism, - - - 290
11. Partial Fracture of the Long Bones in Children, 293
12. Rupture of the Kidney, - - - - 295
13. Cholera at Paris, - - - 298
14. Remarks on the Pathology and Treatment of the Cholera. By J. G. Hopkinson, M. D., - 303
15. Accidents from the admission of Air into the Veins. By J. C. Warren, M. D., - - - 310
16. Letter on Cholera from Professor Chapman, - 315
17. Algid Fever of Smyrna compared with the Cholera, 319
18. Dysentery accompanied with an unusual affection of the Skin, - - - - - - 319
19. Mitchell's Elements of Chemical Science, - - 320

